# A Recombinant Measles Vaccine with Enhanced Resistance to Passive Immunity

**DOI:** 10.3390/v9100265

**Published:** 2017-09-21

**Authors:** Emily Julik, Jorge Reyes-del Valle

**Affiliations:** School of Life Sciences, Arizona State University, Tempe, AZ 85287, USA

**Keywords:** measles vaccine, infant vaccination, maternal antibodies

## Abstract

Current measles vaccines suffer from poor effectiveness in young infants due primarily to the inhibitory effect of residual maternal immunity on vaccine responses. The development of a measles vaccine that resists such passive immunity would strongly contribute to the stalled effort toward measles eradication. In this concise communication, we show that a measles virus (MV) with enhanced hemagglutinin (H) expression and incorporation, termed MVvac2-H2, retained its enhanced immunogenicity, previously established in older mice, when administered to very young, genetically modified, MV-susceptible mice in the presence of passive anti-measles immunity. This immunity level mimics the sub-neutralizing immunity prevalent in infants too young to be vaccinated. Additionally, toward a more physiological small animal model of maternal anti-measles immunity interference, we document vertical transfer of passive anti-MV immunity in genetically-modified, MV susceptible mice and show in this physiological model a better MVvac2-H2 immunogenic profile than that of the parental vaccine strain. In sum, these data support the notion that enhancing MV hemagglutinin incorporation can circumvent in vivo neutralization. This strategy merits additional exploration as an alternative pediatric measles vaccine.

## 1. Introduction

Infection by measles virus (MV) continues to have an important impact on global health and particularly on the health of the very young. Despite the use of safe and effective attenuated vaccines against MV for more than 50 years, this extremely infectious virus still killed approximately 134,200 people in 2015, the great majority of them young children [1,2]. Furthermore, though measles incidence declined drastically over the first four decades of attenuated vaccine use, measles incidence stabilized over the last decade at approximately 20 million cases and 100,000 to 150,000 deaths each year; indeed, the incidence of deaths from measles increased from 2014 to 2015 [3]. While current MV vaccines have a remarkable record of success, part of the continuing impact of MV results from the imperfect effectiveness of current vaccines, at a failure rate of 2–10% [4], failure to implement a two-dose vaccine regimen in all countries [5], and, most importantly, the low reliability of current vaccines in young infants [6].

Infants less than nine to 12 months of age are considered ineligible for current MV vaccination methods due to their poor rates of seroresponsiveness. In countries with high risk of MV transmission, the vaccine is administered at nine months of age, when it induces protective immunity in approximately 85% of recipients, while vaccination is delayed until 12 to 15 months of age in countries at low risk of MV transmission, when it reaches its maximal efficiency of approximately 95% responsiveness in a single dose [7,8]. The primary factor that limits successful vaccination of young infants is their variable retention of passive maternal immunity. This humoral immunity, passed across the placenta and in breast milk, protects against early life infection [9], but soon declines to subneutralizing levels that may interfere with vaccination but not with wild-type MV infection [10,11,12]. Specifically, deficient vaccine responses have been documented in infants with titers of maternal antibodies that, while detectable, fall below the lower limit of protection against wild-type measles infection [12,13]; these infants are thus susceptible to wild-type measles infection, but unable to be efficiently vaccinated. The limited efficiency of current MV vaccines in the very young is especially problematic because the highest fatality and complication rates from MV infection occur in infants less than one year of age, prior to eligibility for vaccination [14]. A next-generation measles vaccine with a higher rate of responsiveness that could be administered to younger infants is thus needed.

Previously, with the goal of developing a more effective measles vaccine for young infants, we developed two recombinant modified MVs in a Moraten-equivalent background with added copies of the full-length (MVvac2-H2) or truncated (MVvac2-Hsol) hemagglutinin (H) cistron. Despite this genetic alteration, both recombinants showed a comparable in vitro replication profile as the parental, Moraten-equivalent strain in two cell lines approved for measles vaccine manufacture [15]. Based upon the results of clinical trials that demonstrated the ability of high titer doses of MV vaccine to overcome maternal antibody interference and induce immunity in young infants [16], but that subsequently demonstrated increased mortality attributed to wild type MV-like immunosuppression in high titer vaccine recipients [17], we hypothesized that our modified MVs with higher relevant H antigen expression might similarly overcome maternal immunity within a safe standard titer dose. For MVvac2-H2 in particular, its enhanced immunogenicity suggested that it could improve responses to vaccination, and its measurable resistance to neutralization by low levels of polyclonal immune serum in vitro suggested the possibility that MVvac2-H2 would remain infectious in the presence of low level neutralizing immunity. While the immunogenicity of MVvac2-Hsol could not be rescued in MV-susceptible mice, we show here that MVvac2-H2 is more immunogenic than the parental Moraten-equivalent virus, MVvac2 [18], in young mice. Compellingly, in the presence of artificially introduced sub-neutralizing anti-MV titers, mimicking those observed in human infants, MVvac2-H2 induced substantially stronger neutralizing antibody responses than MVvac2, which was significantly inhibited. Finally, we document the vertical transfer of anti-MV immunity in the gold standard MV-susceptible mouse strain. In this model of passive immunity MVvac2-H2 also proved more immunogenic than the parental strain. These results warrant further experimentation in a naturally MV-susceptible animal model.

## 2. Materials and Methods

### 2.1. Cells and Viruses

Vero/hSLAM cells served as hosts for growth of viral stocks and for neutralization assays. Methods related to the growth of cells and amplification of recombinant viruses have been detailed elsewhere [15]. MVvac2 [18] has identical amino acid coding capacity to the Moraten and Schwarz vaccine strains and so serves as a current vaccine-equivalent control. MVvac2-H2, a modified MV with increased expression of the H protein, was constructed by inserting an additional His-tagged copy of the full-length H coding cistron into an additional transcription unit downstream of the nucleocapsid (N) cistron in the MVvac2 genomic background. A second modified MV, MVvac2-Hsol, directs the secretion of a His-tagged, soluble form of the H protein.

### 2.2. Mouse Inoculations and Sera Transfers

All experimental procedures were performed according to a protocol approved for the period 25 September 2014–24 September 2017 by the Arizona State University Institutional Animal Care and Use Committee (protocol number 15-1391R). All vaccines were diluted to the appropriate titer in Hanks’ Balanced Salt Solution (HBSS, Mediatech, Manassas, VA, USA) with 1% fetal bovine serum (FBS, Atlanta Biologicals, Flowery Branch, GA, USA) just prior to administration.

Immunogenicity of purified particles in young mice: To determine the immunogenicity of viral particles in young MV-susceptible mice, groups of four to five three-week-old HuCD46Ge-IFNar^KO^ mice [19] were inoculated by the intraperitoneal (i.p.) route with a viral particle dose of 10^3^ TCID_50_. Mice were bled 28 days after inoculation, and sera were separated and heat-inactivated.

Immunogenicity in the presence of artificial passive immunity: To artificially model the passive anti-MV immunity that hinders vaccination of young infants, we introduced dilute MV-immune serum to young HuCD46Ge-IFNar^KO^ mice and subsequently inoculated these mice with MVs. Specifically, strongly neutralizing serum, with an anti-MV neutralization titer of 1:3413, was obtained from a HuCD46Ge-IFNar^KO^ mouse immunized with 10^5^ TCID_50_ MVvac2 vaccine vector doses. Serum was diluted 1:300 in HBSS with 1% FBS for a neutralization potency of approximately 1:10 at administration; this potency was confirmed by neutralization assay. As a control, non-immune serum derived from a naïve HuCD46Ge-IFNar^KO^ mouse was diluted by the same procedure. Five-week-old mice received 500 μL of the diluted anti-MV or the control serum via the i.p. route. One day after artificial introduction of passive immunity, the mice were bled to measure anti-MV passive immunity present at the time of vaccination. Mice were then inoculated by the i.p. route with a 10^5^ TCID_50_ dose of MVvac2 or MVvac2-H2 (seven to eight mice per group), or with vaccine diluent alone (two mice). Sera were obtained from all mice 28 days after inoculation of viruses or mock to determine the immunogenicity of the viruses in the presence of artificial passive immunity. Sera were separated and decomplemented at 56 °C for 1 h.

Time course of anti-MV maternal passive immunity transfer: The time course of anti-MV maternal passive immunity transfer and waning in HuCD46Ge-IFNar^KO^ mice was determined by comparing serum anti-MV neutralization titers of MV-immune dams to those of their two- to six-week-old pups. Five six-week-old females were inoculated with a 10^5^ TCID_50_ i.p. dose of MVvac2 and mated. Anti-MV titers in pup sera were assessed at the indicated time point and expressed both as a raw reciprocal titer and as a ratio compared to the immunity in their dam. Sera were collected from three to six pups per time point.

Immunogenicity in the presence of natural passive immunity: A five-week-old female was inoculated with a 10^3^ TCID_50_ i.p. dose of MVvac2. After confirmation of anti-MV neutralization titer (1:40), the female at nine weeks of age was mated. When pups reached four weeks of age, they were inoculated via the i.p. route with 10^5^ TCID_50_ MVvac2 (four mice) or MVvac2-H2 (five mice), or vaccine diluent alone. Three weeks after inoculation, sera were obtained, separated, and decomplemented.

### 2.3. MV Neutralization Assay

Humoral immunity against measles was assessed by incubating two-fold dilutions of serum with 100 TCID_50_ of virus on Vero/hSLAM cells in a 96-well plate format, as described previously [15,18]. Neutralization titer was determined as the highest dilution of sera affording elimination of infectivity, defined as the absence of syncytia. Titers were reported as the average of a determination made in triplicate.

### 2.4. MV Logarithmic Neutralization Index

We adapted a highly sensitive assay to detect neutralizing immunity against yellow fever virus [20,21] to detect measles neutralization. Sera obtained from artificial passive transfer recipients were pooled and mixed with an equal volume of HBSS with 1% FBS containing 10^6.8^, 10^5.8^, or 10^4.8^ TCID_50_ of MVvac2 and allowed to interact at 37 °C for 1 h. Remaining viral titer after interaction was measured by endpoint dilution assay and expressed as the average log_10_(TCID_50_) of triplicate determinations. The logarithmic difference in titer reduction was expressed by subtracting the average infectivity remaining after interaction with pooled sera from recipients of anti-MV passive immunity from that remaining after interaction with pooled sera from control animals.

### 2.5. Statistical Analyses

**Figure 1:**
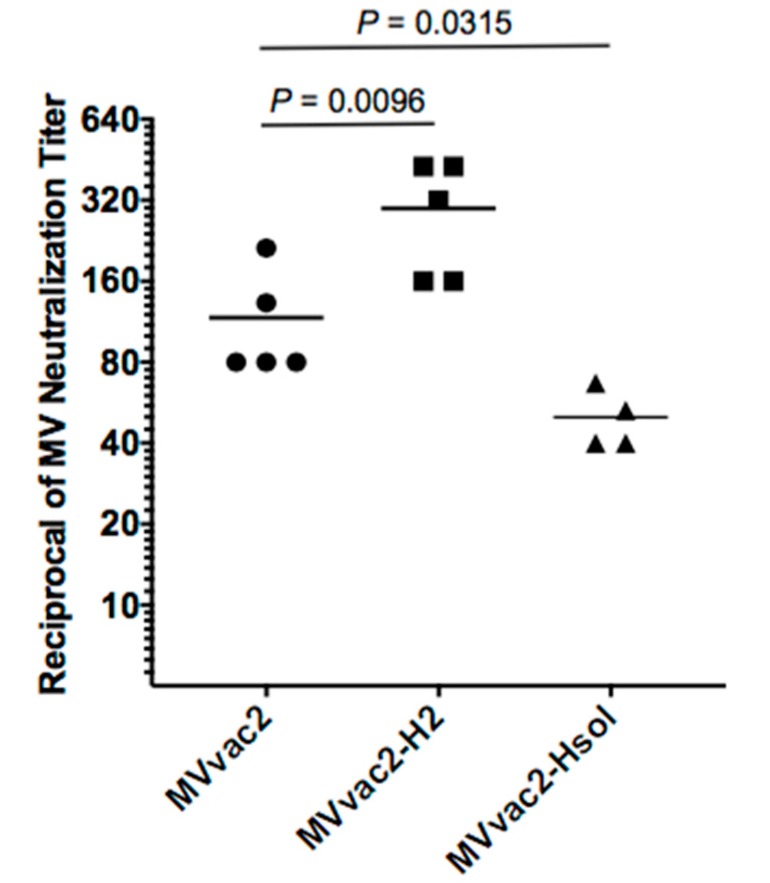
Anti-measles immunogenicity of parental and modified viruses in young MV-susceptible naïve mice. Groups of three-week-old MV-susceptible mice received a single dose of 10^3^ TCID_50_ of purified viral particles of MVvac2, MVvac2-H2, or MVvac2-Hsol. Anti-measles neutralizing immunity was assessed in sera obtained 28 days after vaccination. Each symbol represents one animal and horizontal lines indicate group geometric mean. Statistical significance of differences in neutralizing immunity was assessed by one-way analysis of variance ANOVA followed by Dunnett’s multiple comparisons test using the MVvac2 group as control.

**Figure 2:**
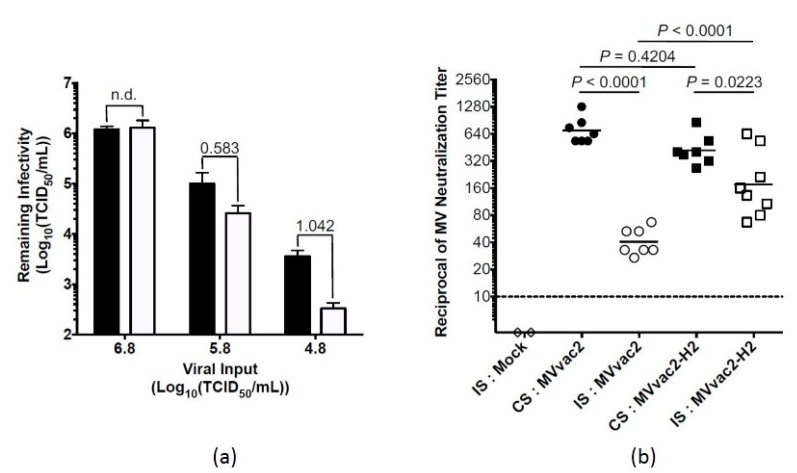
Immunogenicity of MV expressing additional H in the presence of artificially acquired anti-measles antibodies at levels capable of reducing viral titer ex vivo: (**a**) artificially introduced MV-immune serum reduces MVvac2 infectivity ex vivo. Five-week-old MV-susceptible mice were inoculated with equivalent amounts of diluted irrelevant control (black bars) or MV-hyperimmune serum (white bars). Pooled sera obtained from these animals the day after passive transfer was assayed for its impact on MV infectivity after incubation with varying titers of MVvac2. Remaining viral titers following incubation are reported, with bars indicating the standard deviation of three experiments. The logarithmic difference in MV infectivity is numerically annotated above each bar pair, with n.d. indicating no difference; (**b**) anti-MV neutralizing immunity in the presence of artificially introduced neutralizing antibodies. One day after the artificial passive transfer of control serum (CS, black symbols) or immune serum (IS, white symbols), mice from (**a**) were vaccinated with 10^5^ TCID_50_ MVvac2 (circles) or MVvac2-H2 (squares), or mock vaccine (diamonds). Mice were bled 28 days post-vaccination, and anti-MV neutralizing titer was determined. Each symbol represents one animal. Solid horizontal lines represent group geometric mean. The interrupted horizontal line indicates the limit of detection. Statistical significance of differences in neutralizing immunity was assessed by two-way ANOVA on log_2_-transformed reciprocal titers, followed by Sidak’s multiple comparisons test.

Statistical analyses of antibody responses were performed using GraphPad Prism 7 on log_2_-transformed reciprocal neutralization titers. To determine the statistical significance of differences in immune responses of young animals to the modified vaccines (Figure 1), groups receiving MVvac2-H2 and MVvac2-Hsol were compared to the MVvac2 control group using one-way analysis of variance (ANOVA) followed by Dunnett’s multiple comparisons test. To determine the statistical significance of differences in immune responses to MVvac2 or MVvac2-H2 in the presence or absence of artificially transferred passive immunity (Figure 2b), groups were compared using two-way ANOVA followed by Sidak’s multiple comparisons test.

## 3. Results

### 3.1. An MV Incorporating Additional H Is More Immunogenic in Young Mice

Previously, a modified MV with increased expression and incorporation of the full-length H protein, termed MVvac2-H2, proved significantly more immunogenic than the parental vaccine MVvac2 in mature (six- to eight-week-old) outbred, non-susceptible CD1 mice and in mature genetically modified MV-susceptible HuCD46Ge-IFNar^KO^ mice, which express the human CD46 receptor for MV vaccine strains with human-like distribution in a type I interferon knockout background. In both murine hosts, MVvac2-H2 elicited approximately 2.5-fold higher neutralization titers than MVvac2 elicited [15].

To assess the immunogenicity of MVvac2-H2 in the more relevant MV-susceptible mice at a relatively younger age, we inoculated three-week-old HuCD46Ge-IFNar^KO^ mice with a single dose of 10^3^ TCID_50_ purified viral particles. Four weeks later, we documented neutralizing immunity in sera of individual animals by MV neutralization assay (Figure 1). MVvac2-H2 induced neutralization titers 2.5-fold higher than those induced by MVvac2 (geometric mean of 1:108 for MVvac2 vs. 1:272 for MVvac2-H2, *p* = 0.0096, one-way ANOVA) and MVvac2-Hsol (a virus expressing truncated, soluble H protein) induced neutralization titers more than two times lower than those induced by MVvac2 (1:108 for MVvac2 vs. 1:49 for MVvac2-Hsol, *p* = 0.0315). This experiment demonstrates that even relatively young mice make stronger neutralizing immune responses to MVvac2-H2 than to MVvac2.

### 3.2. An MV Incorporating Additional H Is More Immunogenic in the Presence of Artificially Introduced Anti-MV Passive Immunity

Previous work from our group showed that MVvac2-H2 resists anti-MV neutralizing serum in vitro, retaining its infectivity by two orders of magnitude greater than MVvac2 [15]. Based on this observation, we hypothesized that MVvac2-H2 would induce stronger immune responses than MVvac2 would in the presence of passive immunity due to its greater infective stimulus. To test this hypothesis, we developed a model based on the artificial transfer of subneutralizing anti-MV immunity to MV-susceptible mice and their subsequent vaccination. We introduced subneutralizing anti-MV immunity in HuCD46IFNar^KO^ mice by inoculating homologous diluted hyperimmune measles serum to the animals. We then assessed the anti-MV potency of sera obtained from mice just prior to vaccination. As expected, the introduction of antiserum with a calculated potency of 1:10 to the mouse system, where it was further diluted, resulted in serum anti-MV titers that fell below the limit of detection by neutralization assay (<1:4), with the exception of one animal where we were able to document an anti-MV neutralizing titer of 1:10. We next applied a more sensitive measure of anti-MV immunity by assaying the impact of these sera upon MV infectivity ex vivo using a logarithmic neutralization index approach. For yellow fever virus, such an approach has been well documented to correlate with protection [20] and serves as a highly sensitive measure of neutralizing antibodies. As shown in Figure 2a, we observed dose-dependent MV neutralization by sera from passive transfer recipients. Pooled sera from animals that received passive anti-MV immunity reduced MV infectivity up to ten-fold. Together, these data demonstrate that subprotective titers of neutralizing antibodies, similar to those observed in infants during the window of maternal antibody waning, were successfully introduced to the mice.

Having determined that the artificially introduced passive immunity was of sufficient potency to interfere with vaccine infectivity ex vivo, we sought to measure whether this immunity was also sufficient to interfere with vaccine take in vivo and, if so, whether MVvac2-H2 could overcome this interference. The day after administration of passive immunity, mice were bled to obtain serum and then received a single intraperitoneal dose of 10^5^ TCID_50_ MVvac2 (seven mice), MVvac2-H2 (eight mice), or vaccine diluent alone (two mice, indicated by “mock”). We used a high dose to provide a robust infective stimulus. Two additional control groups of seven mice each received diluted non-immune serum the day prior to inoculation with either MVvac2 or MVvac2-H2.

Twenty-eight days after vaccination (Figure 2b), mice inoculated with MVvac2 after transfer of anti-MV artificial passive immunity developed neutralizing titers with a mean 17-fold lower (1:41) than those that received the same vaccine after passive transfer of non-immune serum (1:696), a difference that was highly statistically significant (*p* < 0.0001, Figure 2b). The subprotective neutralizing immunity introduced to the animals to model passive maternal immunity thus strongly interfered with MVvac2 take. Conversely, mice that received MVvac2-H2 after transfer of anti-MV artificial passive immunity developed titers with a mean only 2.4-fold lower than those that received the same vaccine in the presence of the passively transferred irrelevant sera (1:175 in the presence of immune serum versus 1:420 in the presence of control serum, *p* = 0.0223). In the presence of anti-MV immunity, MVvac2-H2 therefore induced significantly stronger, 4.3-fold higher neutralizing titers than MVvac2 did (1:175 vs. 1:41, respectively, *p* < 0.0001). In sum, even low levels of passive anti-MV immunity strongly inhibited the induction of active humoral immunity by MVvac2. This low-level passive anti-MV immunity proved insufficient, however, to significantly interfere with vaccination by MVvac2-H2. Interestingly, in control recipients of passively transferred irrelevant, non-immune serum, MVvac2 induced slightly, though not significantly, higher neutralization titers than MVvac2-H2 (means of 1:696 compared to 1:420, respectively, *p* = 0.4204). We have also observed this trend, not reaching statistical significance, in naïve MV-susceptible mice vaccinated with 10^5^ TCID_50_ (averages of 1:516 and 1:354 for MVvac2 and MVvac2-H2, respectively, *p* = 0.3949). This trend opposes the significantly greater immunogenicity documented for MVvac2-H2 when administered at a lower dose of 10^3^ TCID_50_.

### 3.3. Transfer of Anti-MV Immunity from Dam to Pup and Measles Vaccine Response in Young Mice with Naturally Acquired Passive Immunity

**Figure 3:**
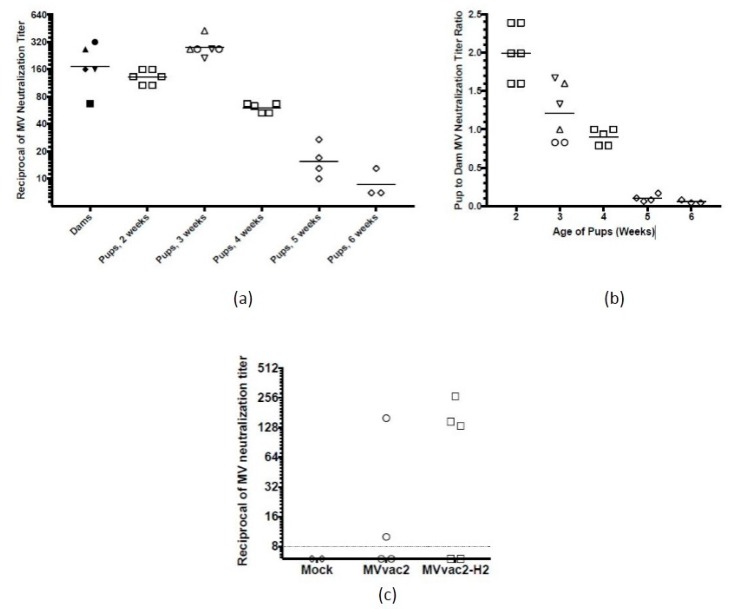
Natural passive transfer of anti-measles maternal immunity and vaccine immunogenicity in its presence: (**a**) time course of passive transfer of anti-MV maternal antibodies in MV-susceptible mice. Five MV-susceptible dams were vaccinated against MV and mated. Their pups were euthanized at the indicated age and serum collected to determine anti-measles status, expressed here as the raw reciprocal titers. Pups (white symbols) from the same litter share the same symbol shape, which they also share with their dam (black symbols), with each symbol representing one animal. Horizontal lines indicate group mean; (**b**) reciprocal titers from (**a**) are also expressed as ratios of pup to dam neutralizing titers; (**c**) parental and modified measles vaccine immunogenicity in the presence of maternally acquired anti-measles antibodies. Four-week-old littermates of a MV-immune dam were vaccinated with 10^5^ TCID_50_ of the indicated virus or mock, and anti-MV neutralizing immunity was assessed three weeks later. Each symbol represents one animal and the interrupted horizontal line indicates the limit of detection.

We sought to evaluate the modified virus in a more physiologically relevant experimental model. Initially, we aimed to detect and quantify maternally derived humoral immunity in pups of MV-immune HuCD46Ge-IFNar^KO^ dams, where immunity was introduced by inoculation with the Moraten vaccine-equivalent MVvac2. As shown in Figure 3a,b, MV-immune HuCD46Ge-IFNar^KO^ dams passively transmit anti-MV humoral immunity to their offspring. Because multiple dams were used, with different anti-MV neutralization titers, we express pup titers both as their raw reciprocal titers (Figure 3a) and as a ratio of those documented in their dams (Figure 3b) to facilitate comparison between pups of different dams. The efficiency of maternal antibody transfer is such that passive immunity accumulated in two-week-old pups to levels almost twice as high as in their dam, with pup neutralization titers an average of 1.97-fold higher than those documented in their dam (Figure 3a, mean of 1:132 in pups and 1:67 in dam). Over the next two weeks of life, passive maternal immunity in pups declined to levels comparable to that in their dam (mean of 1:60 in pups and 1:67 in dam, 0.90-ratio). By six weeks of age, anti-MV neutralizing immunity, although detectable in all pups tested, was deeply reduced to a less than 0.1 proportion from their dam.

Since the passive transfer of anti-MV neutralizing immunity mimics passive transfer in humans, we decided to test the immunogenicity of MVvac2-H2 in four-week-old pups. As shown in Figure 3c, MVvac2-H2 was more immunogenic than the parental strain MVvac2 in young animals with passive anti-MV immunity. Three out of five animals developed a protective anti-measles titer (1:60 to 1:120) after MVvac2-H2 immunization in contrast with only one out of four after MVvac2 immunization.

## 4. Discussion

We show here that, consistent with our previous findings in older mice, young mice respond more strongly to MVvac2-H2 than to its parental current vaccine-equivalent, MVvac2. Remarkably, in the presence of low levels of artificially introduced passive immunity, responses of MV-susceptible mice to MVvac2 are very significantly inhibited. Responses to MVvac2-H2 in the presence of artificial passive immunity are also inhibited, though not nearly as strongly as the responses to MVvac2. The difference in fold inhibition might be partly attributable to the induction of slightly, though not significantly, lower neutralizing titers by MVvac2-H2 than MVvac2 at this 10^5^ TCID_50_ dose in the presence of control non-immune serum. Nonetheless, in the presence of the artificial passive immunity, MVvac2-H2 induced titers more than four-fold higher than those induced by MVvac2, a difference that was highly statistically significant. This leads us to speculate that MVvac2-H2 could better induce active immunity in the presence of maternal antibodies. It remains of interest to us to test the range of MVvac2-H2 resistance to passive immunity in vivo by assaying different levels of artificially introduced neutralizing immunity. In this era of vaccination, when only approximately 40% [22] of infants have detectable maternal immunity by four months of age, and the neutralization titer of such immunity is of a low level correspondent to its waning, we may argue that vaccine performance in the presence of subprotective immunity is the most clinically relevant indicator of potential success in young infants.

Intriguingly, the relatively enhanced immunogenicity of MVvac2-H2 is apparently lost at a high titer (10^5^ TCID_50_) dose in adult MV-susceptible mice as shown in Figure 2b, where control groups of mice that received non-immune serum followed by vaccination with 10^5^ TCID_50_ of MVvac2 or MVvac2-H2 made similar responses. We have observed in an independent experiment that naive adult mice receiving 10^5^ TCID_50_ of MVvac2 or MVvac2-H2 also made similar responses (Julik, E and Reyes-del Valle, J. in preparation). In contrast, in a previous study [15] and in results presented in Figure 1, we documented the higher immunogenicity of MVvac-H2 using a more relevant dose of 10^3^ TCID_50_, which is the same as that mandated for vaccination of humans. We speculate that lower dosages allow parsing of differences in immunogenicity due an optimized distribution volume to inoculum ratio. It remains an open and interesting question how vaccine titers relate between this mouse model and humans.

By documenting that female MV-susceptible HuCD46Ge-IFNar^KO^ mice efficiently transfer anti-MV passive immunity to their pups and testing our modified vaccine in pups of immune dams, we showed the relatively more reliable immunogenicity of MVvac2-H2. In this novel physiological passive transfer model, a greater proportion of MVvac2-H2 recipients compared to MVvac2 recipients developed protective immunity after immunization in the presence of maternal antibodies (3 of 5 vs. 1 of 4 respectively, with an additional animal from the MVvac2 group making a marginally detectable response). Only a small number of animals could be tested, so strong conclusions cannot be drawn from these data. However, these preliminary results intriguingly suggest an “all-or-nothing” response. In the presence of maternal immunity, pups either fail to mount a humoral response to vaccination, as indicated by undetectable neutralization titers of less than 1:10, or mount a robust, protective response, developing neutralization titers comparable to those documented in adult mice or mice lacking passive immunity. A caveat to this interpretation is a single mouse from the MVvac2 group in whom marginally detectable (1:10) anti-MV immunity was measured at the conclusion of the study. We may speculate that this low level immunity reflects waning maternal immunity at seven weeks of age rather than an active immune response to vaccination. While residual maternal immunity was not detected in two littermates who received a mock inoculation, we have documented some variability of maternal antibody retention between littermates (Figure 3a), at least until six weeks of age. Alternatively, MVvac2 take may have been limited, but not entirely prevented, by maternal antibody interference in this mouse. Overall, these results provide reasonable support for our hypothesis that the increased presence of neutralization targets (H protein) on the surface of MVvac2-H2 effectively shifts its threshold for neutralization. MVvac2-H2 is more likely to resist neutralization by maternal antibodies, and thus induces protective immunity in a greater proportion of recipients. As we observed in the artificial passive transfer model (Figure 2b), MVvac2-H2 appears susceptible to a degree to inhibition by passive immunity, demonstrated there by slightly lower antibody titers in the presence of passive immunity and in the natural passive transfer model by a proportion of recipients who fail to respond. Still, MVvac2-H2 performs significantly better than MVvac2, inducing stronger responses and in a greater proportion of recipients than MVvac2 induces. A comparable increase in the proportion of young infant responders to measles vaccination would strongly benefit the drive to reduce measles mortality and eventually eradicate the virus.

HuCD46Ge-IFNar^KO^ mice are already considered the gold-standard small animal model for MV vaccine immunogenicity. There are limitations in studies based upon this model. For instance, infectious virus can only be occasionally recovered from tissues of these mice due to limited replication and thus assessment of viremia, a proxy analysis for in vivo viral fitness, cannot be accurately determined [19]. Such evaluation is possible only in non-human primates in which a comparative analysis of MVvac2 and MVvac2-H2 viral replication profile could be performed reliably. Nonetheless, data obtained from this mouse model of MV immunogenicity was recently corroborated in a Phase I clinical trial [23,24]. In the case of the recombinant viruses that progressed to a clinical trial, initial testing of the vaccine in mice involved inoculation via the i.p. route [23], a route of delivery we also followed here and that is consistent with delivery in our previous study [15], facilitating comparison between those results and the results presented here. The sub-cutaneous (s.c.) route of administration has also been used in recombinant vaccine studies in this mouse model and is the main vaccine inoculation route followed in humans. In our hands, the magnitude of antibody responses to MV vaccination of these mice is similar regardless of whether the vaccine is administered via the i.p. route or the s.c. route at the base of the tail, though it is possible that the route of administration might affect other aspects of the immune response. In this study, we opted to evaluate neutralization responses, which are the correlate of protection against MV infection. While T cell responses are important for viral clearance and recovery from infection, they do not consistently provide protection from infection [25], unlike neutralizing responses. They may, however, modulate the severity of infection and have been relatively easier to achieve in young infants than protective antibody responses [26]. It remains of interest to us to test cellular responses to MVvac2-H2, particularly in the more relevant macaque model, but neutralizing immunity remains the goal of any successful next-generation MV vaccine.

Our results presented in Figure 3 further suggest the utility of HuCD46Ge-IFNar^KO^ mice as a model for preclinical evaluation of experimental pediatric vaccines against measles. Passive transfer of maternal anti-MV immunity has been documented in the cotton rat model of measles infection [27] and in BALB/c mice [28,29]. To our knowledge, we present here the first report of the dam-to-pup transfer of passive maternal immunity to MV in HuCD46Ge-IFNar^KO^ mice. While unsurprising, this result is significant nonetheless, given the proven preclinical utility of this mouse model for testing MV vector vaccine responses. These results suggest the further utility of this model for testing responses to experimental infant-targeted MV vaccines. Certainly, there are known differences in the physiological transfer of passive maternal immunity in mice as compared to humans, such as primary transfer across the placenta in humans versus in breast milk for mice, and a slightly different time course of accumulation and loss in humans compared to mice. Time course studies of human maternal immunity measured by neutralization titer have suggested an average half-life of approximately 47 days [8]. Our data suggest a shorter half-life for the waning of neutralizing maternal immunity in HuCD46Ge-IFNar^KO^ mice of approximately 14 days. Notably, in the cotton rat model where maternal antibody titers were also measured by neutralization assay [27], maternal immunity appears to fade on a similarly more rapid time course. In BALB/c mice, maternal immunity also fades entirely between 35 and 50 days of age when measured by plaque reduction neutralization assay [28], consistent with our observations. Decay extends to ~15 weeks of age when measured by the more sensitive but less clinically relevant anti-MV ELISA in BALB/c mice [29]. In HuCD46Ge-IFNar^KO^ mice, neutralizing passive maternal immunity accumulated in pups to levels at least 1.97-fold higher than in their dams. While it is not clear whether this represents the absolute maximal accumulation of maternal antibodies in MV-susceptible mice, it nonetheless reflects observations in certain human populations, where maternal antibodies accumulate in newborns to levels 1.8-fold higher than in their mothers [22].

In sum, these data show that MVvac2-H2 maintains its enhanced immunogenicity in young MV-susceptible mice and in the presence of low levels of passively transferred anti-MV neutralizing antibodies. These two key observations support our central hypothesis that MVvac2-H2 will perform better than current MV vaccines in young infants. We expect this to prove true both in young infants retaining passive maternal immunity, where MVvac2-H2 may overcome the inhibitory effect of this immunity on vaccine responses, and in young infants lacking maternal immunity, where the enhanced immunogenicity of this vaccine at a standard titer dose will boost protective neutralization responses. We also present a new twist on a proven mouse model for MV vaccination. The passage of neutralizing maternal antibodies in MV-susceptible mice will allow preclinical assessment of the performance of MVvac2-H2 in the presence of passive immunity. It is significant that all of the five countries that account for 77% of global measles incidence in 2015 begin MV vaccination at eight to nine months of age with coverage ranging from 70 to 90% [30]. In two of those countries, only a single dose of MV vaccine is offered, rather that the recommended two-dose regimen, and thus a more reliable alternative able to induce seroconversion in a greater proportion of responders in a single dose would be of value. Furthermore, a recent study suggested that most of measles cases in the US are imported from countries with low measles vaccination discipline, enhancing the potential for a regional outbreak [31]. Optimizing the performance of the current measles vaccination schedule by using a more immunogenic genetically modified option at an earlier age may therefore prove elemental to measles eradication. In sum, these experiments lay additional foundation for future evaluation of MVvac2-H2 in non-human primates, where features like potential reactogenicity and protection from challenge can be assessed in addition to neutralizing immunogenicity.
